# Individualized Prediction of PTSD Symptom Severity in Trauma Survivors From Whole-Brain Resting-State Functional Connectivity

**DOI:** 10.3389/fnbeh.2020.563152

**Published:** 2020-12-21

**Authors:** Xueling Suo, Du Lei, Wenbin Li, Jing Yang, Lingjiang Li, John A. Sweeney, Qiyong Gong

**Affiliations:** ^1^Huaxi MR Research Center (HMRRC), Department of Radiology, West China Hospital of Sichuan University, Chengdu, China; ^2^Research Unit of Psychoradiology, Chinese Academy of Medical Sciences, Chengdu, China; ^3^Functional and Molecular Imaging Key Laboratory of Sichuan Province, West China Hospital of Sichuan University, Chengdu, China; ^4^Department of Psychiatry and Behavioral Neuroscience, University of Cincinnati, Cincinnati, OH, United States; ^5^Mental Health Institute, The Second Xiangya Hospital of Central South University, Changsha, China

**Keywords:** psychoradiology, post-traumatic stress disorder, functional magnetic resonance imaging, resting-state, functional connectivity, connectome-based predictive modeling

## Abstract

Previous studies have demonstrated relations between spontaneous neural activity evaluated by resting-state functional magnetic resonance imaging (fMRI) and symptom severity in post-traumatic stress disorder. However, few studies have used brain-based measures to identify imaging associations with illness severity at the level of individual patients. This study applied connectome-based predictive modeling (CPM), a recently developed data-driven and subject-level method, to identify brain function features that are related to symptom severity of trauma survivors. Resting-state fMRI scans and clinical ratings were obtained 10–15 months after the earthquake from 122 earthquake survivors. Symptom severity of post-traumatic stress disorder features for each survivor was evaluated using the Clinician Administered Post-traumatic Stress Disorder Scale (CAPS-IV). A functionally pre-defined atlas was applied to divide the human brain into 268 regions. Each individual’s functional connectivity 268 × 268 matrix was created to reflect correlations of functional time series data across each pair of nodes. The relationship between CAPS-IV scores and brain functional connectivity was explored in a CPM linear model. Using a leave-one-out cross-validation (LOOCV) procedure, findings showed that the positive network model predicted the left-out individual’s CAPS-IV scores from resting-state functional connectivity. CPM predicted CAPS-IV scores, as indicated by a significant correspondence between predicted and actual values (*r* = 0.30, *P* = 0.001) utilizing primarily functional connectivity between visual cortex, subcortical-cerebellum, limbic, and motor systems. The current study provides data-driven evidence regarding the functional brain features that predict symptom severity based on the organization of intrinsic brain networks and highlights its potential application in making clinical evaluation of symptom severity at the individual level.

## Introduction

There is a high risk for trauma survivors to develop post-traumatic stress disorder (PTSD; Yehuda and Flory, 2007), a highly debilitating psychiatric disorder characterized by symptoms including avoidance of trauma-related stimuli, re-experiencing of the trauma, hyperarousal, and altered cognition and mood (American Psychiatric Association, 2013). Psychoradiology, a new field of radiology, aims to use brain imaging to not only advance understanding of psychiatric disorders but also play a clinical role in diagnostic and treatment planning decisions (Sun et al., 2018; Huang et al., 2019; Gong, 2020). Previous studies have identified brain connectivity network alterations in trauma survivors who develop PTSD compared with those who do not (Patel et al., 2012; Lei et al., 2015; Kennis et al., 2016; Akiki et al., 2018; Niu et al., 2018). However, these alterations were based on group-level comparisons. Thus, it remains unclear whether image data can be helpful for the clinical evaluation of symptom severity in individual trauma survivors.

Functional magnetic resonance imaging (fMRI) is a noninvasive technique assessing neural activity. Functional connectivity analyses, examining associations of activity across different brain regions, have demonstrated robust and unique patterns of brain activity that predict neuropsychological traits and clinical symptoms across individuals (Dubois and Adolphs, 2016; Rosenberg et al., 2018). Modeling the associations between phenotypic measures (e.g., ratings of illness severity) and functional brain organization can provide a basis for establishing the clinical utility of imaging data (Gao et al., 2019).

The comprehensive map of functional connectivity in the human brain is defined as the “functional connectome” (Biswal et al., 2010). Recent research has applied functional connectome analysis to predict a broad range of phenotypic measures, including intelligence (Finn et al., 2015), creativity (Beaty et al., 2018), attention (Rosenberg et al., 2016), cocaine abstinence (Yip et al., 2019), cognitive impairment (Lin et al., 2018), and symptom severity of autism spectrum disorder and attention deficit hyperactivity disorder (Lake et al., 2019). Few studies have attempted to investigate the relationship between functional connectivity and PTSD symptom severity (Lanius et al., 2010; Zhou et al., 2012; Tursich et al., 2015; Zandvakili et al., 2020). Further, most included participants were receiving treatment with psychotropic medications and had psychiatric comorbidities, which may have influenced study findings. Also, some studies used a seed-based method in which findings may have been biased by the particular seed region chosen. While resting-state functional connectivity analyses of symptom severity in PTSD have been informative, the prediction of PTSD symptom severity using the whole brain functional connectome before drug treatment remains to be established in noncomorbid trauma survivors.

In the current study, we applied a recently developed connectome-based predictive modeling (CPM) method (Shen et al., 2017) to identify the neural networks that allow for accurate prediction of individual PTSD symptom severity reflected in CAPS-IV scores in a cohort of trauma survivors using resting-state brain functional connectome features. Clinician-Administered PTSD Scale (CAPS), a widely used structured interview, is considered the gold standard in PTSD research for measuring its severity, and is a rating scale with excellent psychometric properties including strong discriminant and convergent validity, good clinical utility, and sensitivity to clinical alteration (Weathers et al., 2001). There are two neural models when investigating the neuropathophysiology underlying PTSD. One is the traditional neural circuit of PTSD based on studies of fear processing, with critical structures including medial prefrontal cortex, amygdala, and hippocampus (Rauch et al., 2006). The other is the triple network model including central executive, default mode, and salience networks (Patel et al., 2012). Based on prior research, we hypothesized that individual symptom severity would be related to intrinsic functional connectivity across distributed networks, e.g., traditional fear neural circuit or the triple network model.

## Materials and Methods

### Participants

This retrospective study was approved by the Medical Research Ethics Committee of West China Hospital, Sichuan University, and informed written consent was obtained from all participants before the study. One-hundred and twenty-two survivors were recruited between 10 and 15 months after the 2008 Sichuan earthquake event (see Table 1). Inclusion criteria were as follows: (i) physical experience of the earthquake; (ii) without any physical injury; and (iii) personally witness serious injury, death, and/or the collapse of buildings. Exclusion criteria included history of any neurological or psychiatric disorder other than PTSD, psychiatric comorbidities evaluated by the structured clinical interview for DSM IV diagnosis (SCID), pregnancy, history of drug or alcohol abuse, and recent medication that might have an effect on brain function. Each participant was evaluated by using the CAPS-IV as a continuous measure of symptom severity (Blake et al., 1995). Of the 122 traumatized earthquake survivors included, 64 fulfilled diagnostic criteria for current PTSD at the time of fMRI examination. All participants had received no prior treatment with psychiatric medications. fMRI data from these participants have been reported previously elsewhere. In 2014, Gong et al. (2014a) investigated the relationship between resting-state fMRI data and PTSD Checklist scores using a multivariate analytical method, whereas our current work constructed a prediction model at the level of individual patients using the CAPS-IV and a CPM method.

**Table 1:** Demographics and clinical characteristics of the subjects.

Characteristic	Earthquake survivors (*n* = 122)
Age (years)^b^	43.0 ± 10.3 (20–67)^a^
Gender (male/female)	36/86
Years of education^b^	7.3 ± 3.4 (0–16)^a^
Time since trauma (months)^b^	12.2 ± 2.2 (8–15)^a^
Clinician-administered PTSD scale	40.1 ± 22.3 (3–95)^a^

*^a^Data are presented as the mean ± SD (range of minimum–maximum). ^b^Age, years of education, and time since trauma were reported by participants at the time of magnetic resonance scanning*.

### Data Acquisition

Resting-state fMRI is a technique for measuring spontaneous blood oxygen level-dependent (BOLD) fluctuations that reflect resting neurophysiological activity of the brain. The acquisition of fMRI from survivors took place between 10 and 15 months after the earthquake at the same day of clinical assessment. A total of 200 image volumes sensitized to BOLD signal changes were collected for each participant using a 3-T MRI system (GE EXCITE, Milwaukee, WI, USA) equipped with an eight-channel phased array head coil. The fMRI data were obtained with the following scanning parameters: repetition time = 2,000 ms; echo time = 30 ms; field of view = 240 × 240 mm^2^; voxel size = 3.75 × 3.75 × 5 mm^3^; matrix size = 64 × 64; flip angle = 90°; slice thickness = 5 mm, no slice gap; and 30 axial slice per volume. For each participant, each functional run resulted in a total scanning time of 400 s. Each subject was instructed to lie quietly with their eyes closed during the scanning.

### Data Pre-processing

SPM8 software^1^ was used to perform the pre-processing of fMRI image data. First, the original 10 time points were deleted to establish magnetic tissue stabilization. Then, slice timing correction was conducted to correct for intra-volume acquisition delay. The images were further realigned for the correction of head movement. To reduce the influences of head motion, a scrubbing method was performed, which deleted volumes with frame-wise displacement (FD) >0.5 mm. Images were normalized using echo-planar imaging templates (voxel size: 3 × 3 × 3). Subsequently, linear trends in time series were removed. Nuisance signal (including the Friston 24-parameter head motion model, the white matter signal, the cerebrospinal fluid signal, and the global signal) were regressed out. Finally, functional data were linearly detrended and temporally bandpass (0.01–0.1 Hz) filtered to eliminate effects of high-frequency noise and low-frequency drift, and smoothed (Gaussian kernel with a full-width at half-maximum of 4 mm). None of the participants showed excessive head motion (defined as rotation >2°, translation >2 mm, or mean FD >0.15 mm) throughout the course of scans.

### Functional Connectivity

Using the GRETNA^2^ toolbox (Wang et al., 2015), the functional brain network was constructed. The Shen brain atlas was applied to parcellate the brain into 268 region of interest including the cortex, subcortex, and cerebellum (Supplementary Table 1 and Supplementary Figure 1) to define the network nodes (Shen et al., 2013), as in previous CPM work (Rosenberg et al., 2016; Beaty et al., 2018; Lake et al., 2019; Yip et al., 2019). This involved computation of mean time courses for each of the 268 nodes (i.e., average time course of voxels within the node) for use in node-by-node pairwise Pearson’s correlations. The resultant *r* coefficients were transformed using Fisher’s *z*-transformation to create symmetric 268 × 268 connectivity matrices in which each value of the matrix represents the connection strength between all pairs of nodes.

### Connectome-Based Predictive Modeling

CPM was conducted using previously validated and freely available custom MATLAB scripts (Shen et al., 2017). Briefly, CPM took brain connectivity data and behavioral measures (in this case, functional brain connectivity matrices and CAPS-IV scores, respectively) as input to create a linear predictive model of the PTSD symptom severity using the connectivity matrices. Spearman’s correlations with a statistical significance *P*-value threshold of 0.05 were calculated between edge weights and disease symptom measures across the training participants to identify negative and positive predictive networks. According to the suggestion of Shen et al. (2017), the Spearman’s correlation rather than the Pearson’s correlation were calculated as the CAPS-IV scores do not follow a normal distribution. For the positive prediction network, edges were positively associated with the disease symptom measures, and for the negative prediction network, edges were negatively associated with the disease symptom measures. Therefore, elements in the negative and positive prediction network were defined by associations with CAPS-IV scores instead of negative or positive functional connectivity themselves. While both networks were used for predicting the same variable, they were by definition independent, because a single edge was either a negative or a positive predictor. Individual summary values were then calculated by summing the significant functional connectivity strength in each network and were applied to construct linear predictive models to estimate the relationships between network strength with CAPS-IV scores. The resultant polynomial coefficients (including slope and intercept) were then used to predict symptom severity. In the current study, leave-one-out cross-validation (LOOCV) analysis was employed. Briefly, the “left-out” participant’s predicted CAPS-IV score was obtained by the predictive model trained on all other participants’ data iteratively until all participants had a predicted score.

Spearman’s correlations between the predicted and actual CAPS-IV scores were used to assess the model performance. To address the problem of non-independence of analyses in the leave-one-out folds, nonparametric permutation testing rather than parametric testing was performed to evaluate statistical significance. To obtain empirical null distributions for Spearman correlation coefficients, the correspondence between CAPS-IV scores and connectivity matrices were randomly shuffled 1,000 times and the CPM analysis was re-conducted using the shuffled data. The *p*-values for leave-one-out predictions were computed based on the null distributions as previous suggested (Shen et al., 2017).

### Contributing Nodes and Edges in the Prediction of CAPS-IV Scores

To investigate the functional anatomy of the contributing elements, the distribution of nodes and edges were summarized in two methods. First, the 268 nodes were classified into 10 macroscale brain regions that were anatomically defined, including the prefrontal cortex (46 nodes), cerebellum (41 nodes), temporal lobe (39 nodes), limbic cortex (36 nodes), parietal lobe (27 nodes), occipital lobe (25 nodes), motor cortex (21 nodes), subcortical structures (17 nodes), brainstem (9 nodes), and insular cortex (7 nodes; Finn et al., 2015; Rosenberg et al., 2016). Second, the 268 nodes were parcellated into eight canonical networks previously defined using a clustering algorithm (Finn et al., 2015), including medial frontal, motor, subcortical-cerebellum, visual (I, II, and association), frontoparietal, and default mode networks. The number of connections between all pairs of macroscale brain regions or canonical networks was then computed. Last, the number of each node’s connections was used to evaluate their importance (Rosenberg et al., 2016; Beaty et al., 2018). The functional connectivity patterns of the top 10 nodes with the most connections were determined.

### Validation Analyses

The following procedures were performed to further evaluate reproducibility of our results. First, a 1,000-iteration permutation test was used to generate an empirical null distribution of the test statistic. To determine whether our main results depended on the choice of different iterations, we reran the CPM analysis using a 5,000-iteration permutation test. Second, we constructed functional connectomes using another parcellation scheme, the automated anatomical labeling (AAL) atlas (Tzourio-Mazoyer et al., 2002) and repeated the entire analyses. Third, we also performed CAPS-IV score prediction using LIBSVM (Chang and Lin, 2011) to implement support vector machine regression (SVR) with a linear kernel. Positive and negative edges were selected the same way as in CPM, and both positive and negative edges were input into SVR as features. The performance of the SVR algorithm was evaluated using correlation between the observed and predicted values.

### Support Vector Machine (SVM) Analysis

Exploratory SVM analyses were applied to the functional connectivity matrices to determine whether functional networks can detect PTSD patients and trauma-exposed non-PTSD (TENP) controls at the individual level. For full details of SVM evaluation, please refer to our recent study (Lei et al., 2020).

## Results

### Preliminary Analyses

**Figure 1:**
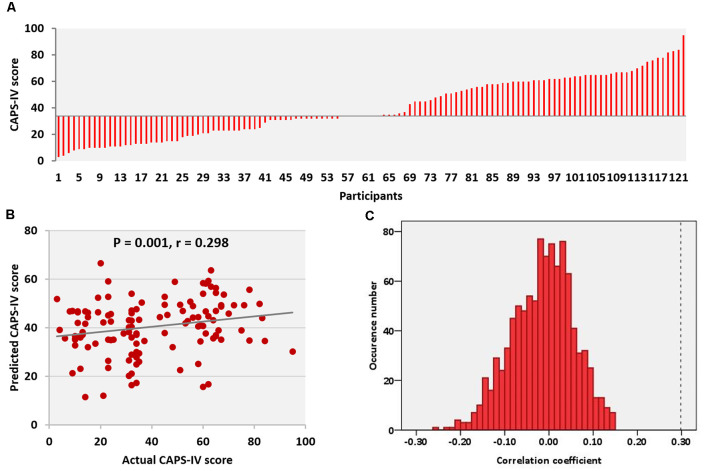
Clinician Administered Post-traumatic Stress Disorder Scale (CAPS-IV) scores and performance of the prediction model. **(A)** CAPS-IV scores across all participants. **(B)** Correlation between actual and predicted CAPS-IV scores. **(C)** Permutation distribution of the correlation coefficient (*r*) for the prediction analysis. CAPS, clinical-administered post-traumatic stress disorder (PTSD) score.

Subjects differed widely in respect to their degree of psychological distress reflected in CAPS-IV scores (Figure 1A). There were no statistically significant correlations between CAPS-IV scores and age (*r* = 0.072, *P* = 0.430) as well as the mean FD head motion (*r* = −0.076, *P* = 0.407). CAPS-IV scores did not differ between genders (*P* = 0.881).

### Predicting CAPS-IV

The relations between connection strength of the positive/negative network and CAPS-IV scores in individual trauma survivors were examined by implementing a LOOCV approach. Model performance was evaluated using Spearman’s rank correlation on predicted and actual scores, and statistical significance was determined using a 1,000-iteration permutation test, repeating the prediction analysis, and determining the fraction of correlations between predicted and actual scores that were as extreme as the original data. Results indicated that resting-state brain functional connectivity in the positive network was related to individuals’ CAPS-IV scores (correlation between actual and predicted scores: *r* = 0.30, *P* = 0.001, permutation test, Figures 1B,C). However, resting-state functional connectivity in the negative network could not reliably predict CAPS-IV scores (correlation between actual and predicted scores: *r* = 0.17, *P* = 0.07).

### Functional Anatomy

**Figure 2:**
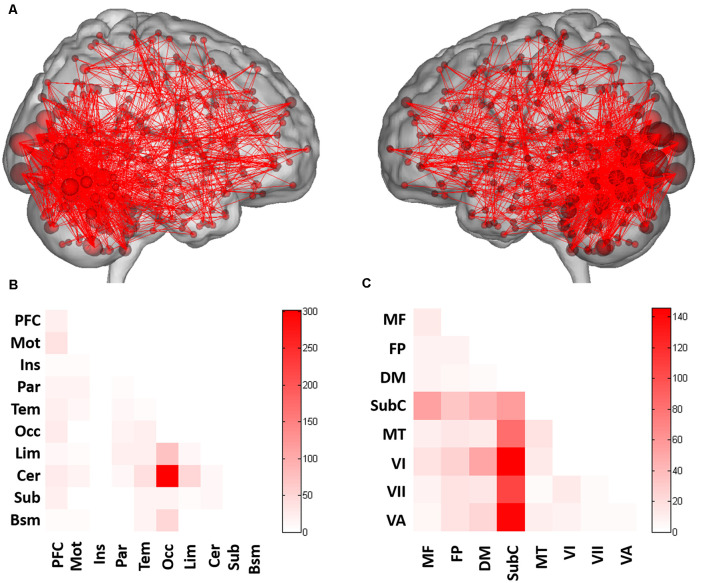
Functional connections predicting CAPS-IV scores. **(A)** Positive (red) networks selected by the prediction model. For the positive network, increased edge weights (i.e., increased functional connectivity) predict higher CAPS-IV scores. **(B)** Connections plotted as number of edges within and between each pair of macroscale regions. **(C)** Connections plotted as number of edges within and between each pair of canonical networks. Cells represent the total number of edges connecting nodes within (and between) each macroscale region or canonical network, with darker colors indicating a greater number of edges. PFC, prefrontal; Mot, motor; Ins, insula; Par, parietal; Tem, temporal; Occ, occipital; Lim, limbic; Cer, cerebellum; Sub, subcortical; Bsm, brainstem; MF, medial frontal network; FP, frontoparietal network; DM, default mode network; SubC, subcortical-cerebellum network; MT, motor network; VI, visual I network; VII, visual II network; VA, visual association network.

Across all folds of LOOCV, 1,006 edges (2.81% of the 35,778 total edges) in the positive prediction network appeared in every iteration of the LOOCV and were defined as the contributing network (Figure 2A). CPM analysis revealed the functional anatomy of networks in which activity was related to CAPS-IV scores. We applied the parcellation that grouped the 268 nodes into 10 macroscale brain regions, which were anatomically defined, to the positive networks to identify connections between macroscale brain regions involved in prediction. Connections between occipital lobe and cerebellum and connections of the limbic lobe with cerebellum and occipital lobe were primary predictors of CAPS-IV score (Figure 2B).

When dividing the 268 nodes into the eight canonical networks previously used in Finn et al. (2015), connectivity based on the number of connections within and between canonical networks for the positive networks is shown in Figure 2C. It was revealed that the positive network included relatively more connections of the subcortical-cerebellum network with visual I, visual II, visual association, and motor networks, and connections within the subcortical-cerebellum network were highly involved in prediction (Figure 2C).

**Figure 3:**
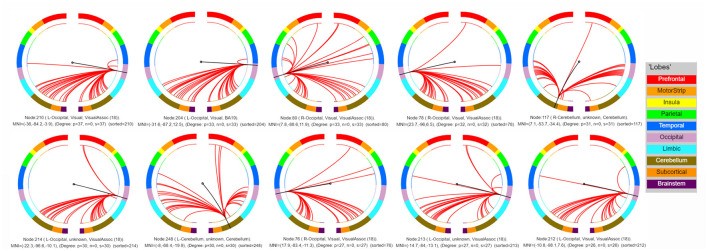
Connectivity patterns of the top 10 nodes with the most connections. L, left; R, right.

Lastly, the top 10 nodes with the most connections were located in the bilateral visual cortex [including bilateral visual association cortex (18) and left visual cortex BA 19] and cerebellum (lobules VI–VII), indicating the critical role of these nodes in predicating the severity of PTSD-related symptoms as reflected in CAPS-IV scores (Figure 3 and Table 2). Note that single-subject levels of CAPS-IV scores were primarily represented by functional connectivity of these regions to other brain regions in addition to their intrinsic connectivity.

**Table 2:** Ten nodes with the most connections selected by the prediction model.

Node	MNI coordinate (mm)			Lobe	Degree
L Visual Assoc (18)	−36	−84.2	−3.9	Occipital	37
L Visual BA 19	−31.6	−87.2	12.5	Occipital	33
R Visual Assoc (18)	7.8	−88.6	11.9	Occipital	33
R Visual Assoc (18)	23.7	−96	6.5	Occipital	32
R Cerebellum	7.1	−53.7	−34.4	Cerebellum	31
L Visual Assoc (18)	−22.3	−96.6	−10.1	Occipital	30
L Cerebellum	−8	−68.4	−19.9	Cerebellum	30
R Visual Assoc (18)	17.9	−83.4	−11.3	Occipital	27
L Visual Assoc (18)	−14.7	−84	−13.1	Occipital	27
L Visual Assoc (18)	−10.8	−98.1	7.6	Occipital	26

*Abbreviation: Assoc, association; L, left; R, right*.

### Validation With Different Schemes

Using different validation schemes, the performance of prediction was re-estimated. The resultant correlation coefficients between actual and predicted CAPS-IV scores remained significant when using 5,000 times permutation test, thus validating the main findings. However, there was no significant prediction in the positive (correlation between actual and predicted scores: *r* = 0.16, *P* = 0.10, permutation tests) or negative network model (correlation between actual and predicted scores: *r* = 0.16, *P* = 0.11, permutation tests) when using the AAL atlas. The application of SVR to the positive networks (correlation between actual and predicted scores: *r* = 0.08, *P* = 0.64, permutation tests) or negative networks (correlation between actual and predicted scores: *r* = 0.10, *P* = 0.56, permutation tests) did not allow quantitative prediction of CAPS-IV scores with statistically significant accuracy.

### Single-Subject Classification of PTSD Patients and TENP Controls Using SVM

Using functional connectivity matrices, the mean balanced accuracy of classification of PTSD vs. TENP was 64.5%, with sensitivity 67.1% and specificity 62.0% (*P* = 0.004). To identify brain regions providing greatest contribution to single-subject classification, the mean absolute value of the weights of the model across the different folds of the cross-validation was calculated. The 10 brain regions with the highest mean values are shown in Supplementary Figure 2. It can be seen that most of the brain regions were cerebellum and visual association regions.

## Discussion

We applied a functional brain network analysis in a recently developed machine-learning approach to use fMRI features to predict clinical severity of PTSD symptoms in a group who had experienced acute major life trauma. We have demonstrated that functional brain connectivity allowed for prediction of single-subject PTSD symptom severity independently of confounding variables (i.e., head motion, gender, age, prior treatment with psychiatric medications, and psychiatric comorbidity). Inter-individual differences in CAPS-IV scores were mainly accounted for by the functional brain connectivity between subcortical-cerebellum, visual, limbic, and motor systems. These observations highlight the importance of brain regions outside the classic traditional fear neural circuit and the triple network model as being important in determining the severity of PTSD.

Our prior study showed that the utilization of a multivariate machine-learning approach known as SVM to structural MRI data provided for the discrimination of traumatized survivors who do and do not fulfil the criteria for PTSD (Gong et al., 2014b). However, this study focused on a binary classification between non-PTSD controls and PTSD patients and neglected the severity of PTSD symptoms as a dimensional illness feature of important clinical significance. A data-driven, whole-brain dimensional analysis centered on single-subject variations instead of binary case–non-case classification may be more helpful for obtaining features related to illness severity (Lake et al., 2019). In addition, we previously used a multivariate analytical approach known as relevance vector regression to the whole-brain fMRI data to predict the clinical scores (Gong et al., 2014a). This current research extended these earlier studies by demonstrating that the symptom severity (CAPS-IV measures) could be significantly and quantitatively predicted from a subject-level’s unique resting-state functional brain connectivity by using the CPM approach. CPM has two appealing aspects compared to the multivariate machine-learning approaches (Shen et al., 2017). First, from a practicable point of view, it is simpler to perform CPM, which requires less skill in machine learning and makes it easier to the general neuropsychiatric imaging investigators for conducting replicable data-driven analyses of the relationships between individual brain imaging data and their phenotypic measures. Second, CPM provides straightforward and clearly interpretable one-to-one mapping back to the original feature space in order that the underlying brain connectivity contributing to the predictive model can be easily determined and visualized.

Our findings reveal that intrinsic functional connectivity across multiple neural systems contributes to predicting individual PTSD clinical measures. Specifically, individual CAPS-IV score was primarily accounted for by intrinsic functional connectivity between bilateral visual cortex and cerebellum. Previous studies with PTSD patients have shown hyperactive function of visual cortex compared with controls, which was positively related to PTSD symptom severity (Zhu et al., 2014, 2015; Neumeister et al., 2017). Hyperactivity of visual cortex may be related to disrupted visual imagery in PTSD and underlie the visual re-experiencing of trauma events (Zhu et al., 2014). The cerebellum, another region that integrates sensory information for sensorimotor control, is recognized increasingly to be implicated in cognitive and emotional processing (Schmahmann and Caplan, 2006). Animal studies have established a role for the cerebellum in fear-conditioning consolidation (Sacchetti et al., 2002). Following more and more neuroimaging research, interest in the cerebellum has increased in PTSD. Hyperactivity of the cerebellum in PTSD was observed, including increased resting-state activity (Bing et al., 2013; Ke et al., 2016), increased blood flow during rest (Bonne et al., 2003), and in response to threat-related stimuli in PTSD (Osuch et al., 2001; Pantazatos et al., 2012). During earthquake imagery, the PTSD group demonstrated activation in the bilateral visual cortex and cerebellum while the control group did not (Yang et al., 2004). Similarly, positive correlations were found between resting-state cerebral perfusion in the cerebellum and visual association cortices and PTSD symptom severity in trauma survivors, in keeping with the between-group analysis (Bonne et al., 2003). In addition, a recent study showed that resting-state functional connectivity of the visual association cortices with cerebellum was increased and correlated positively with PTSD symptomatology (Rabellino et al., 2018). Altogether, the alterations in visual cortex and cerebellum might play a critical role in ongoing visual re-experiencing of trauma events and abnormal emotional processing in PTSD.

We further demonstrated that connections of the limbic lobe with cerebellum and occipital lobe were primary predictors of individual CAPS-IV score. The traditional view of PTSD has been that it is a disorder specific to the fronto-limbic fear circuit. Different from this traditional view, regions outside the fronto-limbic circuit were primarily predictive for the severity of PTSD symptoms in the current study. Our findings were consistent with the subset of studies that found functional and structural alterations between limbic and occipital/cerebellum regions (Chen et al., 2012; Leutgeb et al., 2016; McGlade et al., 2020). For instance, Leutgeb et al. (2016) found altered functional connectivity between the limbic system and cerebellum in violent offenders, suggesting that this circuit may contribute to behavioral perturbations linked to PTSD. Similarly, using voxel-based morphometry method, Chen et al. (2012) found that the gray matter volume in the limbic and occipital lobe of trauma survivors were correlated with their CAPS scores. These studies provided evidence for comparable dysfunction in the corticolimbic circuitry, specifically limbic and occipital and cerebellum connectivity in PTSD. Our analytic approach focusing on current symptom severity indicates that systems outside the fronto-limbic fear circuit are crucial to predict the current symptomatology following exposure to serious life trauma.

Prior studies have suggested that symptomatology of PTSD is related to dysfunction of a triple network model that includes the central executive, default mode, and salience network (Patel et al., 2012; Lei et al., 2015; Kennis et al., 2016; Niu et al., 2018). We therefore hypothesized that alterations in this network model would be related to PTSD symptom severity. Contrary to this hypothesis, using the CPM approach, we found that brain regions outside the triple network (e.g., visual cortex and cerebellum) primarily contributed to an accurate prediction of symptom severity. Additionally, connections between the subcortical-cerebellum and motor network and within subcortical-cerebellum were also revealed as key contributions in the prediction of CAPS-IV scores. With respect to subcortical-cerebellum and motor network connectivity, a diffusion tensor imaging study has reported direct connections between the subcortical (e.g., amygdala) and motor cortices (Grèzes et al., 2014), with a resting-state fMRI study providing evidence of a distinct amygdala-sensorimotor functional network (Thome et al., 2017), which might be related to emotional modulation of subjective sensory experiences as they are used in action planning. Disrupted resting-state functional connectivity between subcortical and motor regions in PTSD might reflect maladaptive somatosensory processing (Thome et al., 2017; Belleau et al., 2020). While large-scale, spatially distributed triple network alterations are relatively well established in patients diagnosed with PTSD, our findings extend these studies by adding to a growing evidence base implicating visual, cerebellar, subcortical, and motor involvement in pathophysiological processes that are associated with symptom severity in PTSD. In particular, the cerebellum has, until recently, been underemphasized in PTSD research and in studies of other psychiatric disorders (Baldaçara et al., 2008). It is noteworthy that with the CPM approach, which is based on correlational associations of MRI features and clinical symptoms, we cannot draw a conclusion that PTSD symptoms were “caused” by one or a few networks. As with other studies (Lake et al., 2019), investigating illness-related biology as a continuous spectrum rather than in terms of categorical definition based on meeting and not meeting criteria for diagnosis can add an important approach for studying brain-behavior associations related to current symptom severity vs. those associated with presence of illness.

Notably, when using functional connectivity to discriminate PTSD patients and TENP controls, the accuracy of classification was 64.5%. Our recent study of PTSD found that large-scale brain networks allowed single-subject classification of patients and healthy controls with higher accuracy as might be expected (average: 89%; Zhu et al., 2020). Furthermore, most of the top 10 brain regions providing the greatest contribution to the classification of PTSD and TENP participants overlapped with the key regions in the prediction of CAPS-IV symptom severity scores: among them were the abovementioned visual and cerebellar regions. Therefore, the current study provides an important step toward data-driven diagnostic assessment in PTSD. Although machine learning is not yet available in day-to-day clinical practice, in light of the urgent clinical need for objective biomarkers in the early stage of the disease, it has the potential to inform the development of diagnostic imaging-based markers.

Finally, using a different parcellation strategy (AAL atlas) and predictive model (SVR), we did not detect a pattern of regional connectivity that showed a significant association with clinical scores. Several issues might contribute to the discrepancy. First, the 268-node Shen functional atlas comprises nodes with more coherent time series and specific functional specificity than those defined by the AAL atlas, which might account for the superior performance of the 268-node parcellation because anatomical boundaries do not always match functional ones (Shen et al., 2013). Additionally, when the number of *a priori* selected regions is very small, the risk of no edges or very few edges being selected within some iterations of cross-validation grows remarkably higher. This could lead to unstable models with poor predictive ability. Second, in the CPM model, all positive/negative features were averaged to create summary statistics, which reduced the variance of the summary statistics compared to the original set of features used in the SVR model (Yip et al., 2019). An alternative possibility is that there is a complex relationship between clinical scores and functional connectivity beyond a simple linear correlation in the SVR model. This might be addressed in future studies with larger sample sizes.

Several limitations of this study need to be acknowledged. First, although there is growing evidence that brain functional connectivity may act as a reliable and objective imaging marker of individuals’ phenotypic measures, CPM has not yet been widely used in clinical research. Also, the extent to which brain functional connectivity reflects transient states vs. stable traits is still unknown. To address this issue and determine the observed pattern as a stable feature of symptom severity, future longitudinal studies will be required. Second, participants included in the current study were following a single type of trauma, which increased the homogeneity of the study sample. Since previous studies have suggested that different types of trauma may have different cerebral deficits (Meng et al., 2014), this leaves open the question of whether the findings observed in our study can be generalized to PTSD caused by other types of trauma. Third, since brain connectivity can be acquired from different MRI modalities (i.e., T1-weighted and diffusion tensor imaging), future work might examine structural change in relation to functional patterns. Fourth, the lack of data from an independent sample precludes us from conducting an external validation analysis, and the generalization of the current findings requires further validation using an independent sample. Fifth, some confounding factors, e.g., childhood/early stress, cannot be excluded in the analysis. Future studies may address these issues.

In summary, this study used a recently developed data-driven method to provide evidence that the resting-state brain functional connectivity can reliably and effectively predict individual PTSD clinical scores of trauma-exposed survivors. The significant contribution of visual cortex, cerebellum, limbic, and motor region connectivity to individual PTSD symptom severity indicates that more brain features beyond the triple network model of PTSD need to be considered to comprehensively understand the illness, and the traditional view that PTSD is a psychiatric disorder specific to the fronto-limbic fear circuit may require reconsideration. The current data-driven approach provides a novel tool to characterize the neural underpinning of PTSD severity and might have potential applications to inform the evaluation of subjects in a clinical setting.

## Data Availability Statement

The datasets generated for this study are available on request to the corresponding author.

## Ethics Statement

The studies involving human participants were reviewed and approved by West China Hospital of Sichuan University. The patients/participants provided their written informed consent to participate in this study.

## Author Contributions

QG contributed to conception and design of the study. XS, DL and LL organized the database. XS, DL, WL and JY performed the data preprocessing and statistical analysis. XS wrote the manuscript. DL, WL, JS and QG edited the manuscript. All authors contributed to the article and approved the submitted version.

## Conflict of Interest

JS is a consultant for VersSci. The remaining authors declare that the research was conducted in the absence of any commercial or financial relationships that could be construed as a potential conflict of interest.

## Footnotes

^1^http://www.fil.ion.ucl.ac.uk/spm

^2^http://www.nitrc.org/projects/gretna/
