# Supplementary material for: Individualized Prediction of PTSD Symptom Severity in Trauma Survivors From Whole-Brain Resting-State Functional Connectivity
Source: Front Behav Neurosci. 2020 Dec 21;14:563152. doi: 10.3389/fnbeh.2020.563152 (PMC7779396; doi:10.3389/fnbeh.2020.563152)
Supplement: Supplementary file 1 [file Data_Sheet_1.pdf]

Supplementary Material

Table S1. Subcortical-cerebellum regions of 268-node functional atlas.

Node	MNI coordinates			Lobe
	X	Y	Z	
Right cerebellum	32.2	-78.5	-40.4	Cerebellum
Right cerebellum	6.1	-50.7	-12.3	Cerebellum
Right cerebellum	39.1	-74.9	-29.7	Cerebellum
Right cerebellum	13.7	-40.1	-25.4	Cerebellum
Right cerebellum	23.5	-35.9	-43.0	Cerebellum
Right cerebellum	6.9	-67.9	-37.8	Cerebellum
Right cerebellum	7.1	-69.0	-20.9	Cerebellum
Right cerebellum	46.0	-46.5	-42.4	Cerebellum
Right cerebellum	16.2	-47.2	-52.3	Cerebellum
Right cerebellum	23.4	-59.3	-52.1	Cerebellum
Right cerebellum	21.1	-54.8	-23.8	Cerebellum
Right cerebellum	11.7	-84.1	-34.5	Cerebellum
Right cerebellum	19.6	-73.5	-50.0	Cerebellum
Right cerebellum	36.7	-57.1	-32.8	Cerebellum
Right cerebellum	23.0	-71.8	-29.1	Cerebellum
Right cerebellum	7.6	-56.7	-50.8	Cerebellum
Right cerebellum	41.9	-64.0	-49.2	Cerebellum
Right cerebellum	7.1	-53.7	-34.4	Cerebellum
Right cerebellum	33.8	-47.2	-53.9	Cerebellum
Right cerebellum	30.4	-36.4	-31.1	Cerebellum
Right caudate	21.2	-36.4	22.6	Subcortical
Right caudate	12.7	12.9	11.5	Subcortical
Right caudate	13.7	-4.2	20.9	Subcortical
Right caudate	12.6	20.2	-0.7	Subcortical
Right putamen	26.6	6.3	0.1	Subcortical
Right putamen	14.0	8.3	-9.5	Subcortical
Right thalamus	10.5	-26.8	-2.2	Subcortical
Right thalamus	12.3	-27.7	13.5	Subcortical
Right thalamus	5.5	-9.7	5.2	Subcortical
Right brainstem	4.7	-37.2	-53.0	Subcortical
Right brainstem	9.7	-18.7	-30.7	Subcortical
Right brainstem	6.0	-22.2	-42.3	Subcortical
Right brainstem	6.3	-24.9	-17.5	Subcortical
Right brainstem	7.5	-34.2	-37.3	Subcortical
Left cerebellum	-6.5	-66.2	-37.7	Cerebellum
Left cerebellum	-8.7	-50.6	-39.6	Cerebellum
Left cerebellum	-37.0	-52.9	-31.1	Cerebellum

Left cerebellum	-8.7	-55.2	-52.1	Cerebellum
Left cerebellum	-21.2	-70.0	-48.9	Cerebellum
Left cerebellum	-40.3	-74.2	-29.1	Cerebellum
Left cerebellum	-30.2	-80.2	-40.3	Cerebellum
Left cerebellum	-19.3	-46.4	-53.2	Cerebellum
Left cerebellum	-6.5	-50.2	-11.4	Cerebellum
Left cerebellum	-27.8	-36.0	-30.9	Cerebellum
Left cerebellum	-42.6	-63.7	-46.3	Cerebellum
Left cerebellum	-10.3	-81.7	-32.3	Cerebellum
Left cerebellum	-8.0	-68.4	-19.9	Cerebellum
Left cerebellum	-34.6	-50.3	-54.0	Cerebellum
Left cerebellum	-22.7	-57.9	-48.8	Cerebellum
Left cerebellum	-10.3	-37.7	-25.1	Cerebellum
Left cerebellum	-46.4	-46.8	-42.9	Cerebellum
Left cerebellum	-26.3	-69.5	-30.5	Cerebellum
Left cerebellum	-21.2	-53.4	-23.5	Cerebellum
Left cerebellum	-6.6	-54.7	-26.3	Cerebellum
Left cerebellum	-24.3	-37.8	-44.3	Cerebellum
Left caudate	-10.8	23.9	9.6	Subcortical
Left caudate	-12.5	11.6	8.7	Subcortical
Left caudate	-10.4	10.9	-8.1	Subcortical
Left caudate	-14.6	-3.5	21.1	Subcortical
Left putamen	-24.8	5.6	-0.1	Subcortical
Left thalamus	-9.6	-25.4	-1.4	Subcortical
Left thalamus	-4.9	-10.3	5.8	Subcortical
Left thalamus	-11.6	-25.6	14.8	Subcortical
Left brainstem	-5.0	-21.5	-15.8	Subcortical
Left brainstem	-4.5	-38.0	-53.1	Subcortical
Left brainstem	-7.2	-33.0	-39.4	Subcortical
Left brainstem	-6.1	-18.9	-36.8	Subcortical

MNI, Montreal Neurological Institute.

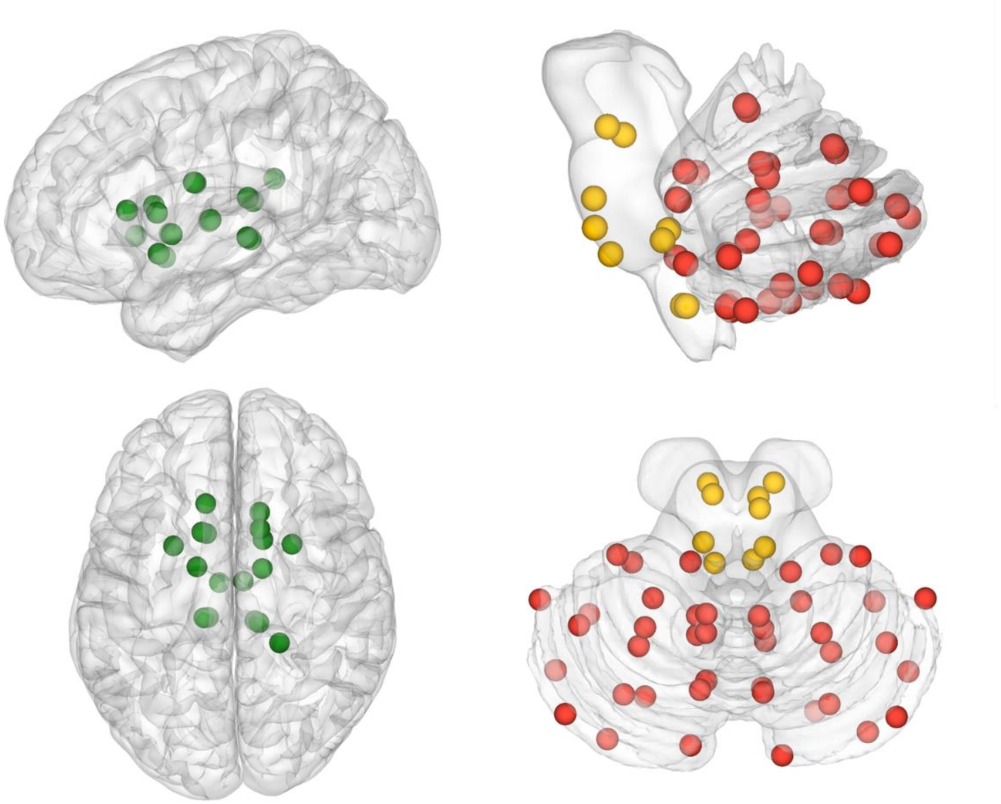

Figure S1. Basal ganglia, brainstem and cerebellum regions of 268-node functional atlas. The nodes were mapped onto the cortical surfaces by using the BrainNet Viewer package (http://www.nitrc.org/projects/bnv). The basal ganglia regions were presented in green, brainstem in yellow, and cerebellum in red.

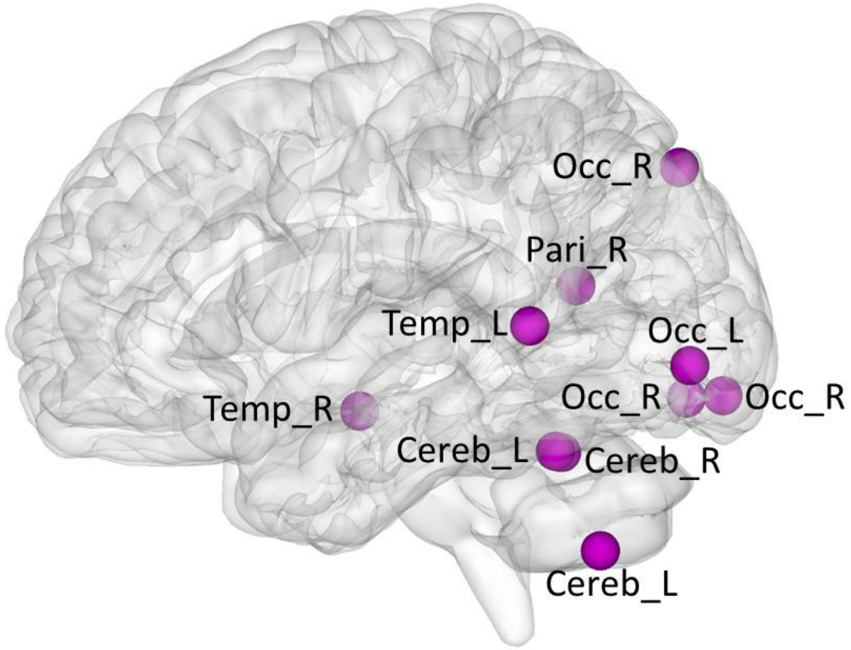

Figure S2. Regions providing the greatest contribution to single-subject classification of PTSD patients and trauma-exposed non PTSD controls. The nodes were mapped onto the cortical surfaces by using the BrainNet Viewer package (http://www.nitrc.org/projects/bnv). PTSD, posttraumatic stress disorder; L, left; R, right; Occ, occipital; Temp, temporal; Pari, parietal; Cereb, cerebellum.
